# P- Hydroxybenzyl Alcohol Alleviates Oxidative Stress in a Nonalcoholic Fatty Liver Disease Larval Zebrafish Model and a BRL-3A Hepatocyte *Via* the Nrf2 Pathway

**DOI:** 10.3389/fphar.2021.646239

**Published:** 2021-04-12

**Authors:** Jing An, Lijun Cheng, Liping Yang, Nali Song, Ju Zhang, Kejian Ma, Ji Ma

**Affiliations:** ^1^ Central Laboratory, Yunnan Institute of Traditional Chinese Medicine and Materia Medica, Kunming, China; ^2^ School of Traditional Chinese Pharmacy, China Pharmaceutical University, Nanjing, China; ^3^ Zhao Tong University, Zhaotong, China; ^4^ Yunnan Key Laboratory for Dai and Yi Medicines, Yunnan University of Chinese Medicine, Kunming, China; ^5^ Key Laboratory of Medicinal Chemistry for Nature Resource Under Ministry of Education, School of Chemical Science and Technology, Yunnan University, Kunming, China

**Keywords:** non-alcoholic fatty liver disease, P-hydroxybenzyl alcohol, zebrafish, oxidative stress, Nrf2-HO-1 pathway

## Abstract

Nonalcoholic fatty liver disease (NAFLD) is the most common chronic liver disease, and it has gradually become the main disease burden in the world. However, the pathogenesis of NAFLD is complex, involving such things as dyslipidemia, oxidative stress, inflammation, etc. This brings to the table a significant challenge for drug development, and there is still no drug approved by the FDA on the market to treat the disease. GAS and HBA are active ingredients of the *orchidaceae* plant *gastrodia elata* and have exhibit effects in ameliorating nervous system diseases caused by oxidative stress. HBA is a metabolite of GAS that could perform lipid regulation and improve oxidative stress on HCD-induced NAFLD larval zebrafish, as reported by previous studies; we therefore explored the role of HBA in lipid regulation and oxidative stress on HCD-induced NAFLD larval zebrafish *in vivo* and FFA-induced BRL-3A hepatocyte *in vitro*. The gene expression of lipogenesis, inflammation, and oxidative stress were measured to investigate the underlying mechanism of HBA, and the potential protein target of HBA was explored by immunofluorescence. Altogether, our data highlight the role of HBA in improving NAFLD by use of its lipid-lowering and anti-oxidative properties *via* the Nrf2/HO-1 signaling pathway, providing a potential therapeutic compound for NAFLD treatment.

## Introduction

Non-alcoholic fatty liver disease (NAFLD) has become a major global concern in recent years; it has a prevalence of 25.24% and is related to the obesity epidemic (Younossi et al., 2016). Currently, NAFLD is considered to be the most common cause of chronic liver disease, defined as the progression of liver disease caused by triglyceride (TG) accumulation (with more than 5% of hepatocyte cytoplasm-present lipid droplets), known as noninflammatory isolated steatosis, and the developed of a more aggressive form of liver disease associated with inflammatory changes and varying degrees of liver fibrosis (Bedossa, 2017; Brunt et al., 2015). However, because of the complex pathogenesis of NAFLD, such as dyslipidemia, insulin resistance, oxidative stress, inflammation, and fibrosis, drug development sees great challenges. (Hirsova et al., 2016; Trauner et al., 2010).

As the “second hit” of the early theory of the “Two hits theory” on NAFLD, oxidative stress and inflammation accelerate the development of NAFLD (Day and James, 1998; Day, 2002; Schuppan and Schattenberg, 2013). During liver injury, functional biomolecules and cells are damaged by unregulated production of free radicals and/or ROS generation, and this leads to pro-inflammatory gene expression (Xu et al., 2019). Antioxidant and anti-inflammatory therapies have been considered to be beneficial in liver diseases. The transcription factor nuclear factor erythrocyte 2-related factor 2 (Nrf2) is the master regulator of the antioxidant response system, anti-inflammatory and cytoprotective properties, and dysregulation of Nrf2 activity and has been revealed to correlate to the development of chronic inflammatory diseases, including NAFLD (Alam et al., 1999; Sandra et al., 2017; Paulina et al., 2018; Tian et al., 2018). Under acute or chronic oxidative stress and inflammatory conditions, Nrf2 is released from Keap-Nrf2 protein components and translated into the nucleus to modulate the gene expression of cytoprotective proteins and enzymes, such as SOD, GST, HO-1, which decreases ROS levels, inflammation, and cell death (Tian et al., 2018; Dinkova-Kostova and Abramov, 2016). The nrf2 pathway plays a crucial role in the pathophysiology of NAFLD mainly by regulating oxidative stress. A previous study showed that activation of Nrf2 could ameliorate NAFLD through reducing oxidation and inhibiting the JNK pathway through treatment with osteocalcin and by improving NASH and hepatic fibrosis in a NASH model by suppressing ERs (Du et al., 2016; Sharma et al., 2018), which proved that Nrf2 could be a potential target for NAFLD.

**FIGURE 1:**
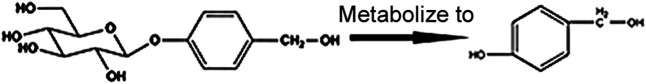
Chemical structure of GAS and HBA as well as metabolization of GAS into HBA *in vivo*.

P- Hydroxybenzyl Alcohol (HBA) and GAS are both the active ingredients of the Orchidaceae plant *gastrodia elata,* which is an herbal agent widely used for the treatment of headaches, tetanus, and inflammatory diseases in East Asian countries (Kim et al., 2011). Pharmacokinetics studies have shown that HBA is a metabolite of GAS in plasma (Figure 1) (Nepal et al., 2019). Previous studies have shown that HBA plays a protective role against oxidative damage-related diseases, such as ischemic brain injury, and coronary heart diseases by scavenging free radicals, such as superoxide and hydroxyl radical (Liu and Akitane, 1993). HBA could also improve neuronal injury by reducing oxidative stress and inflammation through targeting Nrf2 (Ding et al., 2019) and reducing H_2_O_2_-induced PC12 cell death through induced nuclear translocation of Nrf2 (Kam et al., 2011), given that Nrf2 could be a potential target of HBA so as to reduce oxidation and inflammation. Moreover, a previous study has reported that GAS could reduce blood lipids and liver TG and improved NAFLD by way of anti-oxidation in a larval zebrafish model (Geng et al., 2013; Peng et al., 2015; Owais et al., 2019). Combined with the HBA, which is the metabolite of GAS in plasma, we can assume that HBA may have a role in improving NAFLD, and the mechanism may alleviate oxidative stress by targeting Nrf2.

In conclusion, the present study aimed to investigate the efficacy and mechanism of HBA on the treatment of NAFLD. We used an NAFLD larval zebrafish model by feeding HCD *in vivo* and using a BRL-3A cell line treated with Free fatty acid (FFA) *in vitro* to conduct the efficiency investigation. Meanwhile, potential mechanisms of HBA were further investigated by measuring the level of gene and protein expression by immunofluorescence.

## Materials and Methods

### Reagent

Cholesterol (92.5%) and 2′,7′-dichlorofluorescein diacetate (DCFH-DA) were purchased from Sigma-Aldrich (St. Louis, United States). Pioglitazone (98%), Nile Red (95%), Oleic acid, and Palmitic acid were purchased from Aladdin (Shanghai, China). Dulbecco’s Modified Eagle Medium (DMEM) and Fetal Bovine Serum (FBS) were purchased from Gibco (Grand Island, NY, United States). Trypsin was purchased from Solarbio (Beijing, China). BSA-Fatty Acid Free was purchased from Yeasen (Shanghai, China). Penicillin-Streptomycin was purchased from Beyotime (Shanghai, China). HBA (analytical standard, purity >99%) was obtained from National Institutes for Food and Drug Control of China (Beijing, China). Anti-Nrf2 antibody (PA5-27882) was purchased from Invitrogen (Carlsbad, CA, United States). Goat Anti-Rabbit IgG H&L (FITC) secondary antibody (ab6717) was purchased from Abcam (Cambridge, MA, United States).

### Preparation of High-Cholesterol Diet, Free Fatty Acid Solutions, and Drug Solutions

The primary food for Larval zebrafish (AP100) was purchased from Zeigler (PA, United States). The high-cholesterol diet (HCD) was prepared by mixing cholesterol with basic food. The final concentration of cholesterol in HCD was 5% (w/w). A total of 20.296 mg oil acid and 8.547 mg PA were dissolved with 2 ml 0.1 M NaOH in a 75°C water bath as a 50 mM FFA stock solution. We added 2 ml of 50 mM FFA stock solution to 8 ml 10% Fatty Acid Free BSA dropwise at a 55°C water bath to dissolve into 10 mM mother liquid, and this was percolated with 0.22 μM filter membrane. For drug solutions, due to the low solubility of Pioglitazone (PIG) in water, DMSO was used to dissolve the drugs first, followed by dilution in DMSO solution with water to achieve a final drug concentration of 1 μM/L (DMSO 0.001% v/v) for the administration of zebrafish. HBA was directly dissolved in water to achieve a final drug concentration of 20 mg/L, and each group was administrated with DMSO to the same concentration of 0.001% (v/v) in the zebrafish experiment and solved in PBS; this was then diluted by DMEM to a concentration of 20 μm to treat the BRL-3A cell.

### Maintenance of Larval Zebrafish and Treatments

**FIGURE 2:**
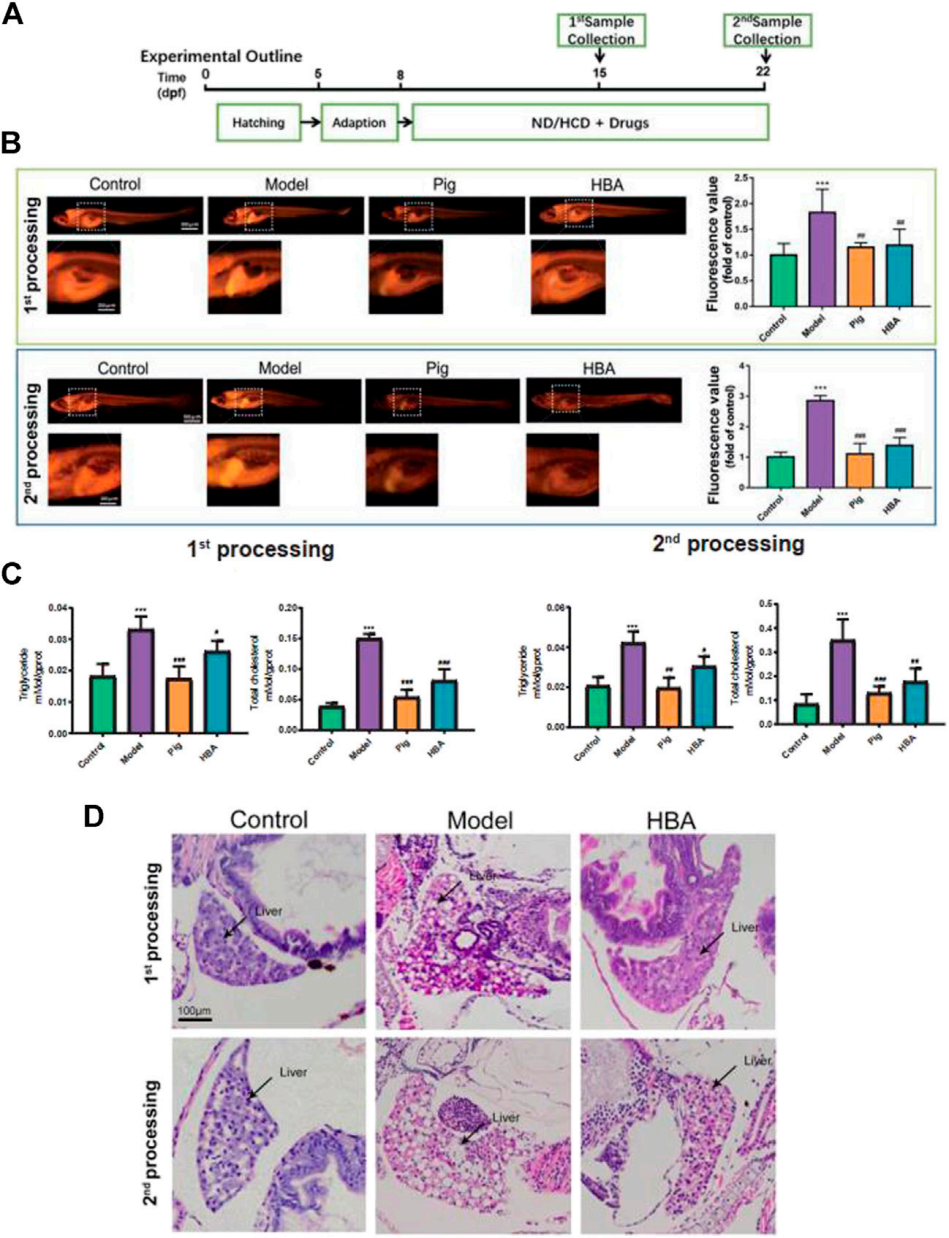
Effect of HBA in regulating lipid metabolism on an HCD-Induced Larval Zebrafish Model in two processes. **(A)** Experimental outline of the larval zebrafish experiment *in vivo*. **(B)** Nile red stain of larval zebrafish and fluorescent quantitation. **(C)** Triglyceride (TG,mmol/gprot) and total cholesterol (TC,mmol/gprot) of larval zebrafish. **(D)** Hematoxylin and eosin (HE) staining of larval zebrafish liver, macrovesicular steatosis, and the differences mentioned with black arrows. Bar indicate means ± SD. ***p < 0.001 represent compared with the control; #*p* < 0.05, *##p* < 0.01, *###p* < 0.001 represent compared with Model. *p* < 0.05 was considered as statistically significant calculated by One-way ANOVA followed by Tukey’s test (*n* = 3, n indicates the replicates of experiment).

The zebrafish embryos were generated by natural spawning from parent wild-type AB-line adult zebrafish. After three days of adaption from 5-days post-fertilization (dpf), larval zebrafish were randomly divided into eight groups in two processes (*n* = 100 for each group). The groups of the first process were as follows: 1) Control group, fed with a Normal diet (ND) for 7 days; 2) HCD group, fed with HCD (20 mg/tank per day) for 7 days; 3) PIG groups, fed with HCD (20 mg/tank per day) and PIG (1 μM) for 7 days; 4) HBA (20 mg/L) group, fed with HCD (20 mg/tank per day) and HBA (20 mg/L) for 7 days. In the second process, the groups were as follows: 1) Control group, fed with a Normal diet (ND) for 14 days; 2) HCD group, fed with HCD (20 mg/tank per day) for 14 days; 3) PIG groups, fed with HCD (20 mg/tank per day) and PIG (1 μM) for 14 days; 4) HBA (20 mg/L) group, fed with HCD (20 mg/tank per day) and HBA (20 mg/L) for 14 days. The dose of HBA treatment was administered according to the result of the survival rate, as seen in Supplementary Material Figure S1A *.* All the groups were maintained following the schedule showed in Figure 2A.

All the animal experiments were approved by the Science and Technology Department of Jiangsu Province and followed the Jiangsu Provincial standard ethical guidelines for the use of experimental animals under the ethical committees mentioned above (SYXK SU 2018–0,019, May 14, 2018).

### BRL-3A Cell Culture and Treatments

*Rattus norvegicus* hepatocellular cell line BRL-3A (American Type Culture Collection, Manassas, VA, United States) was routinely maintained at 37°C in a humidified atmosphere of 5% CO2 in DMEM supplemented with 10% fetal bovine serum (FBS) and 1% penicillin-streptomycin. For Nile red staining, ROS level measured, and immunofluorescent staining, BRL-3A cells (2 × 10^5^) were seeded in 60 mm plates until 70% confluent. Meanwhile, BAL-3A cells were treated with 1 mM FFA for 24 h for the model group, treated with 1 Mm FFA combination with 20 μM HBA for 24 h for the HBA group, and control cells were treated with BSA and NaOH in final concentrations of 1% and 0.2 μM, respectively. For q-PCR, BAL-3A cells (1 × 10^6^) were seeded in 100 mm plates until 70% confluent, and they were then treated as other experiments. The dose of HBA treatment on BRL-3A cell was in accordance with the result of MTT, and the maximum non-toxic dose was applied in Supplementary Material Figure S1B.

### Biochemical Measurement

A total of 30 larval zebrafish of each group were collected; to measure the level of TG, TC, MDA, SOD, ROS, and HO-1, the whole Larval zebrafish were used to prepare the tissue homogenate with PBS in an ultrasonic cell homogenizer (SCIENT2-ⅡD), and we then obtained supernatant by centrifuge. The protein concentration of the tissue homogenate was measured by a BCA protein Quantitative Kit (BeyoTime, China). Triglyceride (TG) levels, total cholesterol (TC) levels, malondialdehyde (MDA) concentration, and superoxide dismutase (SOD) activity were measured by commercial assay kits (Jiancheng, Nanjing, China) following the manufacturer’s instructions. Quantitation of reactive oxygen species (ROS) was detected by Reactive Oxygen Species Assay Kit (BeyoTime, China) following the manufacturer’s instructions, and the fluorescence value read by a microplate reader, which represented the level of ROS. HO-1 level was measured by a Fish Heme oxygenase 1 (HO-1) Elisa Kit (Jiancheng, Nanjing, China). All quantitations of the above kits were read by a multifunctional microplate reader (BioTek, United States).

### Fluorescence Photography

Nile red is a lipophilic fluorescence material that can stain the TG and fatty acid. It can be detected at 543 nm (excitation wavelength) and 598 nm (scattering light). DCFH-DA is an indicator of ROS. DCFH-DA do not have any fluorescence property; only when ROS is oxidizing, it will perform fluorescence property by its oxidized product. A total of 15 Larval zebrafish were incubated with 0.5 μg/ml Nile red or 10 μM/L DCFH-DA (diluted with embryo water) for 30 min at 28.5°C separately. The larval zebrafish were washed with water three times and kept into CMC-Na (4%) on a glass slide for fixation. After treated with FFA with or without HBA 24 h, the medium was removed, and BRL-3A cells were washed with PBS twice then incubated with 0.5 μg/ml Nile red. The fluorescence stereoscope (Olympus SZX16) was immediately used to observe and capture the lipid. All captures were taken with the same parameters (exposure time, ISO, and aperture) between different groups for comparison, and all the above procedures were carried out in the dark. The fluorescence intensity is measured by ImageJ: in brief, the images were converted to an 8-bit grey-scale map, and we adjusted the threshold under the same limited field and selected “limit to threshold” under analyze menu where the mean value represents the fluorescence intensity.

### Immunofluorescence Staining of BAL-3A Cell

BRL-3A cells were treated with FFA with or without HBA 24 h, medium was removed, and BRL-3A cells were washed with ice PBS twice. Then cells were fixed in 4% paraform in ice for 15 min and incubated with a blocking solution containing 5% BSA and 0.3% Triton X-100 for 1 h at room temperature. After blocking, cells were incubated with Nrf2 (1:200, Invitrogen, Thermofisher) overnight in 4°C. After washing, cells were incubated for 20 min with FITC secondary antibody at 37°C and stained with DAPI to label cell nuclei. The signals were visualized, and digital images were obtained by the fluorescence stereoscope (Olympus SZX16).

### Real-Time Quantitative Polymerase Chain Reaction (qRT-Polymerase Chain Reaction) Analysis

A total of 30 liver tissues were separated from larval zebrafish in stereoscope and about 3 × 10^6^ cells of each group were sacrificed for the extraction of total RNA using Trizol reagent (Invitrogen, United States). Reverse transcription was performed by HiScript II qRT SuperMix (Vazyme, China) for the synthesis of cDNA. The qPCR was performed on the StepOnePlus Real-Time PCR System (Applied Biosystems, United States) by adding the ChamQTM universal SYBR qPCR Master Mix (Vazyme, China) and following the manufacturer’s protocol. The specific sequences of primers used in this study were synthesized by General Biotech Co., Ltd. (Shang Hai, China) and are shown in (Table 1). The 2^-∆∆Ct^ method was used to calculate the expression levels of each targeted mRNA normalized to GAPDH.

**TABLE 1:** Specific sequences of primers used in qRT-PCR.

Gene name	Acceccion number (*Danio* rerio)	Forward primer (5’->3′)	Reverse primer (5’->3′)
*Danio rerio*			
srebf1	NM_001,105,129	CAT​CCA​CAT​GGC​TCT​GAG​TG	CTC​ATC​CAC​AAA​GAA​GCG​GT
Fasn	XM_005,169,478	ATC​TGT​TCC​TGT​TCG​ATG​GC	AGC​ATA​TCT​CGG​CTG​ACG​TT
Pparab	NM_001,102,567	CGT​CGT​CAG​GTG​TTT​ACG​GT	AGG​CAC​TTC​TGG​AAT​CGA​CA
Pparg	NM_131,467	CTG​CCG​CAT​ACA​CAA​GAA​GA	TCA​CGT​CAC​TGG​AGA​ACT​CG
Tnfa	NM_212,859	GCT​TAT​GAG​CCA​TGC​AGT​GA	TGC​CCA​GTC​TGT​CTC​CTT​CT
il1b	NM_212,844	TGGCGAACGTCATCCAAG	GGA​GCA​CTG​GGC​GAC​GCA​TA
il6	NM_001,261,449	AGA​CCG​CTG​CCT​GTC​TAA​AA	TTT​GAT​GTC​GTT​CAC​CAG​GA
keap1	NM_182,864.2	CCA​ACG​GCA​TAG​AGG​TAG​TTA​T	CCT​GTA​TGT​GGT​AGG​AGG​GTT
nrf2	NM_182,889.1	TTG​TCT​TTG​GTG​AAC​GGA​GGT	CTC​GGA​GGA​GAT​GGA​AGG​AAG
HO-1	NM_001,127,516.1	GCT​CAA​CAT​CCA​GCT​CTT​TGA​GG	GAC​AAA​GTT​CAT​GGC​CCT​GGG​A
*Rattus norvegicus*			
srebf1	NM_001,276,708.1	ACT​GCT​GTA​AAG​ATG​TAC​CCG​TCC​G	GGC​ACT​GGC​TCC​TCT​TTG​ATT​CC
fasn	NM_017332.2	CTT​TGT​GAG​CCT​CAC​CGC​CAT	ATG​CCA​TCA​GGT​TTC​AGC​CCC
keap1	NM_057152	TGC​TCA​ACC​GCT​TGC​TGT​ATG	CCA​AGT​GCT​TCA​GCA​GGT​ACA
nrf2	NM_031789.2	TTG​TAG​ATG​ACC​ATG​AGT​CGC	TGT​CCT​GCT​GTA​TGC​TGC​TT
HO-1	NM_012580.2	GTA​AAT​GCA​GTG​TTG​GCC​CC	ATG​TGC​CAG​GCA​TCT​CCT​TC

Specific sequences of primers used in this study are shown in the table.

### Statistical Analysis

All the data are expressed as mean ± SD. Graph Pad PRISM (Graph Pad Software, United States) was used for comparing the treatment group and corresponding control by One-way ANOVA followed by Tukey’s test for the significant difference. The differences between groups were considered statistically significant at *p*-value <0.05.

## Results

### Effect of P- Hydroxybenzyl Alcohol in Regulating Lipid Metabolism on High-Cholesterol Diet-Induced Larval Zebrafish Model

To explore the role of HBA in isolated steatosis and mild nonalcoholic steatohepatitis, larval zebrafish models were studied at 7 and 14 days, respectively, by the inducement of HCD, as in a previous study (Ji et al., 2019) (Figure 2A). The effect of HAB in regulating lipid metabolism was measured by Nile Red staining, which is a red phenoxazine lipid fluorescent dye that can label the neutral lipid properties (Greenspan and Fowler, 1985). As the Nile Red results have shown, the intensity of Nile Red fluorescence within the model group increased gradually in the whole trunk and liver over time by the inducement of HCD compared with the Control. Whole-body fluorescent values counted by FIJI also saw extremely significant increases in model groups. However, the increase in Red fluorescence intensity and area were reversed by Pioglitazone and HBA, respectively, in two processes. Notably, the effect is more prominent in the second process (Figure 2B). Furthermore, the level of triglyceride (TG) and total cholesterol (TC) of larval zebrafish were also measured. In the first process, HCD increased the level of TG (0.033 ± 0.004 mMol/gprot, 2.358X to Control) and TC (0.150 ± 0.007mMol/gprot, 3.846X to Control) in the model group. However, both the level of TG and TC in the PIG group (TG: 0.017 ± 0.004 mMol/gprot, 0.515X to Model; TC: 0.054 ± 0.012 mMol/gprot, 0.360X to Model) and HBA group (TG: 0.026 ± 0.003 mMol/gprot, 0.788X to Model; TC: 0.081 ± 0.019 mMol/gprot, 0.540X to Model) were decreased. In the second processing, HCD increased the level of TG (0.042 ± 0.006 mMol/gprot, 2.100X to Control) and TC (0.350 ± 0.087 mMol/gprot, 4.160X to Control), and the level of TG and TC in both PIG group (TG: 0.020 ± 0.005 mMol/gprot, 0.476X to Model; TC: 0.130 ± 0.028 mMol/gprot, 0.377X to Model) and HBA group (TG: 0.030 ± 0.005 mMol/gprot, 0.714X to Model; TC: 0.177 ± 0.055 mMol/gprot, 0.506X to Model) were decreased. The results indicate that HBA has a lipid-regulating effect on HCD-induced larval zebrafish in a manner similar to PIG. To test the role of HBA in hepatic steatosis and steatohepatitis on HCD-induced NAFLD larval zebrafish, the HE staining was conducted in whole larval zebrafish (Figure 2D). In the first process, HCD induced 40–60% of the lipid area in the liver, and lipid accumulation in the liver was almost inhibited by HBA. In the second process, the macrovesicular steatosis almost occupied over 90% of the liver and presented rare inflammation. Notably, HBA also decreased the lipid area in the liver. In conclusion, our results suggested that HBA exhibits lipid regulation and improvement of NAFLD on HCD-induced isolated steatosis even though on a mild nonalcoholic steatohepatitis larval zebrafish model.

### Effect of P- Hydroxybenzyl Alcohol in Oxidative Stress on High-Cholesterol Diet-Induced Larval Zebrafish Model

**FIGURE 3:**
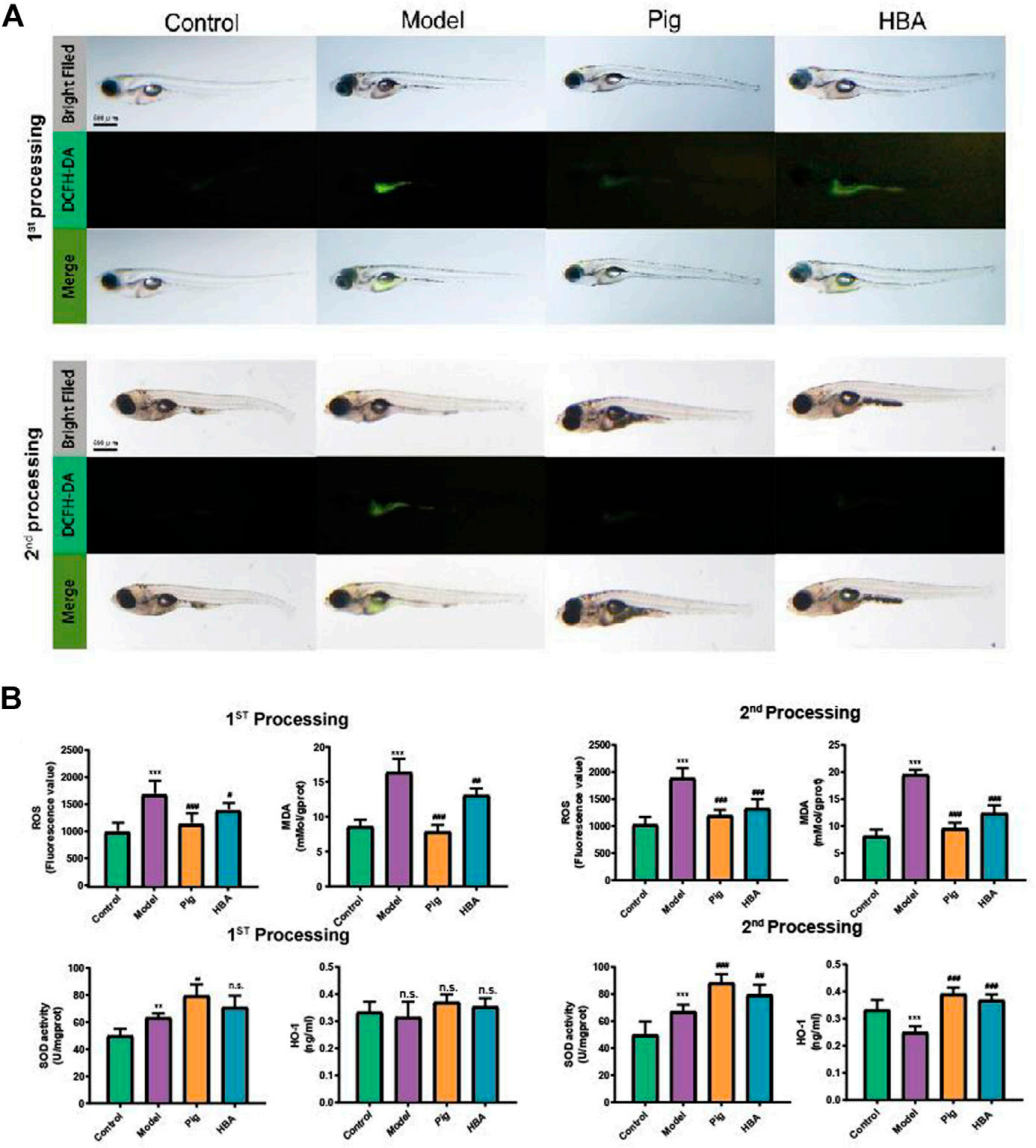
Effect of HBA in oxidative stress on HCD-Induced Larval Zebrafish Model in two processes. **(A)** The ROS production showed in fluorescence image by DCFH-DA staining and merged with a light field image. **(B)** The ROS (Fluorescence value) and MDA concentration of each treated larval zebrafish group; The antioxidase SOD (U/gprot) and HO-1 level (ng/ml) of each treated larval zebrafish group. Bars indicate means ± SD. n. s. indicate no significant; **p < 0.01***p < 0.001 represent compared with the control; #*p* < 0.05, ##*p* < 0.01, ###*p* < 0.001 represent compared with Model. *p* < 0.05 was considered as statistically significant calculated by One-way ANOVA followed by Tukey’s test (*n* = 3, n indicates the replicates of the experiment).

Lipid-modulating oxidative stress accelerates the development of NAFLD, and oxidative stress is also a prominent phenomenon in NAFLD. To reveal the anti-oxidative stress effect of HBA on an HCD-induced oxidant attack on larval zebrafish, 2′,7′-dichlorofluorescein diacetate (DCFH-DA), a fluorescent dye of ROS, was used to stain the oxidant stress product ROS on larval zebrafish. In the two processes, compared to the control group, HCD induced an increase in the fluorescence intensity, and it was distributed observably in the abdomen of larval zebrafish. PIG weakened the ROS-positive area and fluorescence intensity in both processes. However, the role in decreasing the ROS level of HBA is more remarkable in the second process compared with the first process (Figure 3A). ROS and MDA are all cytotoxicity products of lipid peroxidation. Meanwhile, HCD induced a higher level of ROS and MDA in all processes compared with control. Both PIG and HBA reversed the condition HCD induced (Figure 3B). In the first process, HCD increased the level of ROS (1706.0 ± 225.2, 1.7X to Control) and MDA (16.340 ± 1.932 mMol/gprot, 1.901X to Control). However, both the level of ROS and MDA in the PIG group (ROS: 1,157.0 ± 174.1 mMol/gprot, 0.7X to Model; MDA: 9.551 ± 1.080 mMol/gprot, 0.585X to Model) and HBA group (ROS: 1,410.0 ± 115.5, 0.8X to Model; MDA: 12.390 ± 1.445 mMol/gprot, 0.758X to Model) are decreased. Next, we measured the activity of antioxidase SOD and the level of antioxidase HO-1. The results (Figure 3C) showed that HCD did not disrupt the role of SOD (63.410 ± 2.963 U/mgprot, 1.268X to Control) and HO-1 (0.315 ± 0.057 ng/ml, 0.946X to Control) in the first processing. Intriguingly, the level of SOD in the PIG group (79.560 ± 8.41 0U/mgprot, 1.255X to Model) and HBA group (71.130 ± 8.434 ng/ml, 1.121X to Model) has increased, while the HO-1 level has not. In the second process, HCD also increased the activity of SOD (67.200 ± 5.000 U/mgprot, 1.347X to Control). Meanwhile, the level of SOD in the PIG group (88.46 0 ± 6.333 U/mgprot, 1.316X to Model) and HBA group (79.490 ± 7.269 U/mgprot, 1.183X to Model) has increased. Interesting, HCD decreased the level of HO-1 (0.250 ± 0.022 ng/ml, 0.749X to Control), while the level of HO-1 in PIG group (0.391 ± 0.024 ng/ml, 1.564X to Model) and HBA group (0.369 ± 0.021 ng/ml,1.476X to Model) has increased. These results indicate that HBA has the effect in regulating oxidative stress on HCD induced Larval Zebrafish by regulating multiple antioxidase.

### mRNA Expression in Liver Changes P- Hydroxybenzyl Alcohol on the Larval Zebrafish Model

**FIGURE 4:**
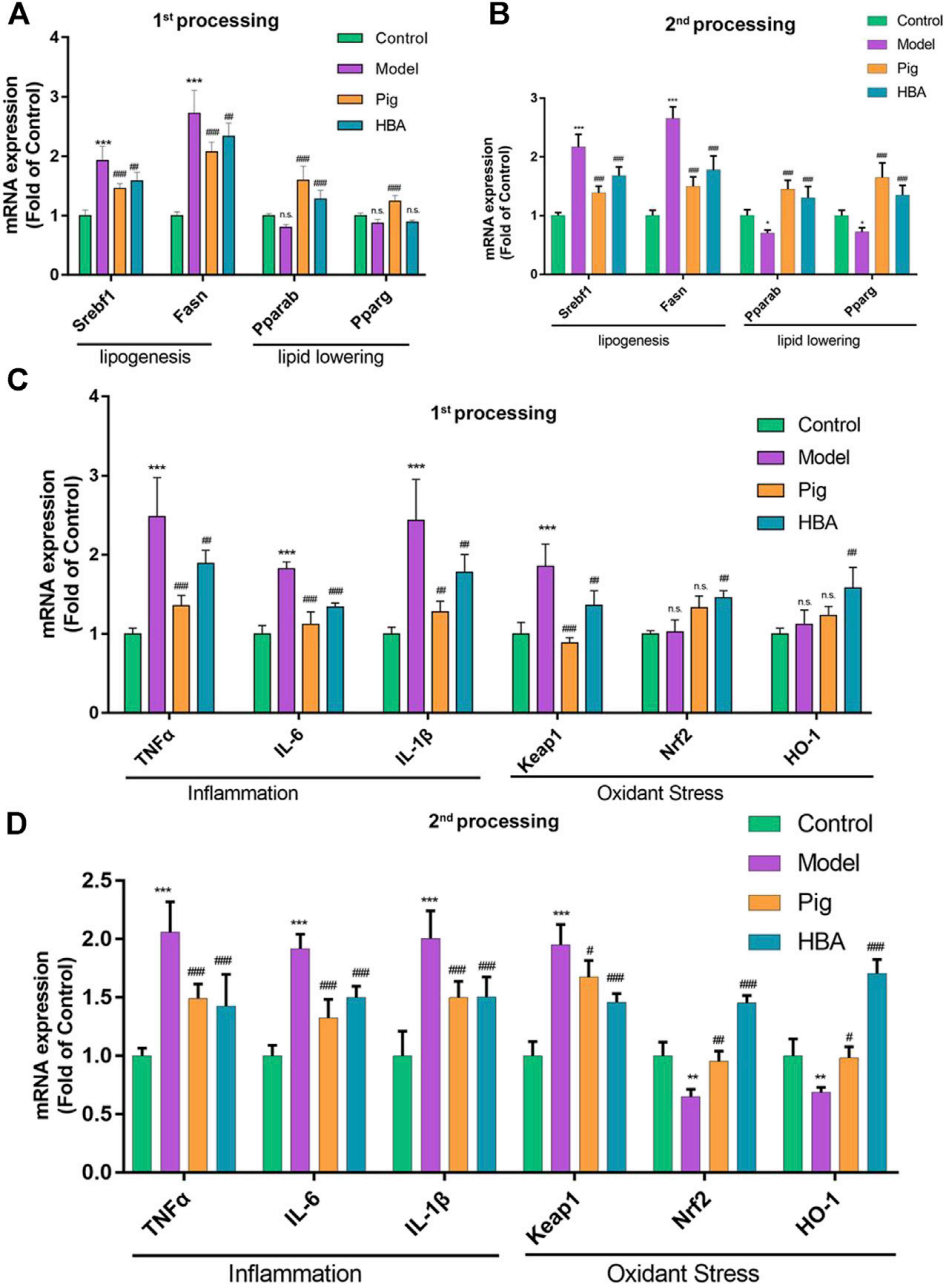
Liver mRNA Expression Changes HBA on the Larval Zebrafish Model. **(A,B)** the gene expression level of lipogenesis and lipid-lowering of each treated larval zebrafish group in the first process B in the second process. **(C,D)** the gene expression level of inflammation and oxidant stress of each treated larval zebrafish group in the first process D in the second process. Bars indicate means ± SD. n. s. indicate no significant; *p < 0.05**p < 0.01***p < 0.001 represent compared with the control; #*p* < 0.05, ##*p* < 0.01, ###*p* < 0.001 represent compared with Model. *p* < 0.05 was considered as statistically significant calculated by One-way ANOVA followed by Tukey’s test (*n* = 3, n indicates the replicates of the experiment).

HBA exhibits an effect on lipid regulating and anti-oxidative stress on HCD-induced NAFLD larval zebrafish. Thus, we further explored the underlying mechanism of HBA. The gene expression levels of *srebf1, fasn, pparα, pparγ* (related to lipid metabolism), *tnfα, il-6, il-1β* (related to inflammation), *keap, nrf2,* and *ho-1* (related to oxidative stress) in the liver were measured. As the results suggest, ed lipogenesis-related genes *srebf1* and *fasn* were significantly increased after HCD induction compared with control. Notably, the related gene expression noted above was reduced in the PIG and HBA groups in all processes (Figures 4A,B). However, the mRNA expression of the lipid-lowering related genes *pparα* and *pparγ* decreased in HCD-induced Larval Zebrafish at 14 days and was restored by PIG and HBA, while the change by HCD was induced within 7 days of processing has no significance (Figures 4A,B). Consistently, q-PCR results demonstrated that the inflammation-related gene expression was increased by HCD in after both 7 and 14 days of processing, while the inflammation-related gene expression decreased in both the PIG and HBA groups. Moreover, Nrf2 pathway gene expression did not differ between model and control in short-term HCD treatment but decreased following long-term HCD intervention. Notably, HBA restored the expression of Nrf2-pathway related gene expression as PIG did (Figures 4C,D).

### The Effect of P- Hydroxybenzyl Alcohol on Free Fatty Acid-Induced BRL-3A Cell *In Vitro*

**FIGURE 5:**
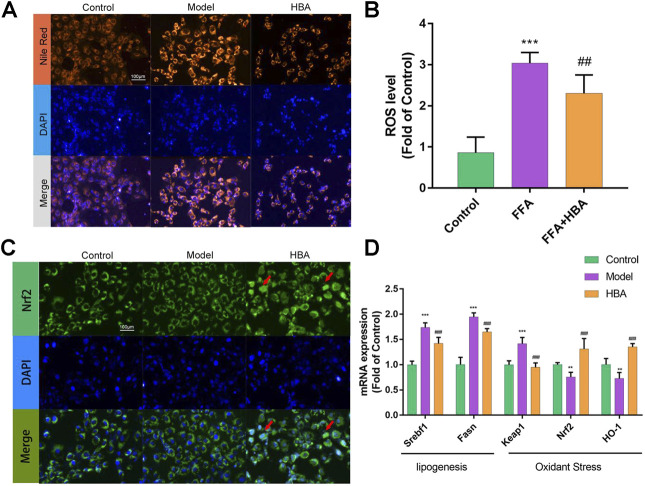
The effect of HBA on FFA-induced BRL-3A cell *in vitro* **(A)** Nile red staining of BRL-3A cells after 1 mm FFA with and without HBA (20 μM) treatment to reveal lipid level. **(B)** Measurement of ROS level in BRL-3A cells after 1 mm FFA with and without HBA (20 μM) treatment. **(C)** Fluorescence image of FITC-conjugated secondary antibody staining indicates the location of Nrf2 (green), DAPI staining indicates the location of the nucleus (blue), and the merged images show the nuclear location of Nrf2 protein. Red arrows show the HBA-induced translocation of Nrf2 to the nucleus. **(D)** mRNA expression profile of HBA on FFA induced BRL-3A cell related to lipogenesis and oxidant stress. Bar indicate means ± SD. **p < 0.01***p < 0.001 represent compared with the control; ##*p* < 0.01, ###*p* < 0.001 represent compared with Model. *p* < 0.05 was considered as statistically significant calculated by One-way ANOVA followed by Tukey’s test (*n* = 3, n indicates the replicates of the experiment).

Based on the results of the investigation into HCD-induced NAFLD Larval Zebrafish, we further explored the role of HBA in lipid regulation and oxidative stress by BRL-3A cells induced with FFA *in vitro*. FAs flow into the liver could induce lipid accumulation and lipotoxicity in hepatocytes. BRL-3A hepatocytes were induced by 1 mm FFA 24 h with or without HBA (20 μM), performed with Nile red staining to measure lipid level, and stained with DAPI to locate the cell. Fluorescence intensity in the cytoplasm increased by FFA, and HBA reduced the fluorescence intensity (Figure 5A). Treatment with 1 mM FFA (62.740 ± 2.986, 3.551X to Control) increased the fluorescence intensity, and HBA (45.370 ± 6.068, 0.723X to Model) decreased the fluorescence intensity (Supplementary Material Figure S2A). The ROS level in hepatocytes was measured, as rising lipid levels in hepatocytes could induce lipid peroxidation and produce ROS. As the results show in Figure 5B, FFA increased the ROS level, and HBA significantly reduced the ROS level. The results implied that HBA also plays a role in lipid-lowering and anti-oxidative stress in hepatocytes. Moreover, we investigate the intrinsic mechanism through immunofluorescence staining combined with q-PCR. The immunofluorescence staining result indicated that HBA facilitates Nrf2 nuclear translocation, unlike both the control and model groups (Figure 5C). Furthermore, the expression of the lipogenesis-related gene and Nrf2 pathway gene were estimated by way of q-PCR. The results showed that FFA could increase the expression of the lipogenesis-related gene and decrease the expression of the Nrf2 pathway gene. Notably, HBA decreased the expression of lipogenesis-related gene and increased the expression of Nrf2 pathway gene (Figure 5D). The results suggested Nrf2 may be the potential target of HBA in regulating oxidative stress on NAFLD.

## Discussion

There are still no drugs on the market for NAFLD that have been approved by the FDA owing to its complicated pathogenesis. An imbalance between TG synthesis and utilization leading to TG accumulation is likely to be the first step in the pathophysiology of NAFLD (2). The development of peripheral IR and hyperinsulinemia contributes to the increased FA influx to the liver. Inflammation develops when the influx of FAs toward the liver, leading to reactive oxygen species (ROS) formation, increased production of inflammatory cytokines, ER stress, and hepatocellular dysfunction and injury in a process named lipotoxicity. These can all ultimately result in the development of NASH (Hirsova et al., 2016; Trauner et al., 2010). The animal models of NAFLD (rodents) are time-consuming and exhibit large individual differences. In recent years, zebrafish has become a widely used disease model due to its characteristics of having 87% genome similarity with humans, being lucid, and its availability for mass rearing (Ji et al., 2019). A previous study (Ji et al., 2019) discovered that feeding larval zebrafish with HCD for 7 days could induce noninflammatory isolated steatosis, and feeding with HCD for 14 days could induce mild nonalcoholic steatohepatitis; meanwhile, the level of ROS increased gradually. Further studies have proven that the larval zebrafish is a suitable model for NAFLD research and screening the potential medicine for NAFLD (Ma et al., 2018). Therefore, the larval zebrafish was induced by HCD for 7 and 14 days in the present study to explore the role of HBA in isolated steatosis and nonalcoholic steatohepatitis respectively *in vivo*. Moreover, the effects on FFA intake and FFA-induced lipotoxicity in hepatocyte BRL-3A *in vitro* were further used to investigate the efficiency and mechanism of HBA.

HBA and GAS are active compounds of *Gastrodia elata,* which is an ancient clinical herb that is widely used in China for disorders of the central nervous system. GAS played a role in lowering lipid in serum and exhibiting hepatic lipid-lowering effects, improving oxidation in an NAFLD larval zebrafish model as previous research reported (Geng et al., 2013; Peng et al., 2015; Owais et al., 2019). HBA is a main metabolite of GAS, and HBA has the same effect on nerve injury disease with GAS through ameliorating oxidative stress and inflammation; however, there is some research about the role of HBA on lipid metabolism and liver disease (Kim et al., 2011). Thus, to explore the role of HBA in lipid-lowering and anti-oxidation in different disease progressions of NAFLD, larval zebrafish were induced by HCD for 7 or 14 days *in vivo*. The level of whole-body lipid in larval zebrafish was measured by Nile red staining, which is a fluorochrome staining neutral lipid (Greenspan and Fowler, 1985). Lipid accumulation in the liver was demonstrated by HE staining. The result (Figure 2B) suggested that the lipid level in larval zebrafish increased by HCD feeding for 7 and 14 days and increased gradually following time HCD treating. HBA could reduce the rise of Nile Red fluorescence intensity coupled with the whole body TG and TC level induced by HCD in two progressions; it was identified that HBA exhibits positive treatment effects as pioglitazone (Figures 2B,C). Liver HE results showed the lipid accumulation was more severe in the second progression, presenting with more macrovesicular lipid droplets and mild inflammation. With HBA interference, lipid in the liver was reduced in two stages and reduced the size of the liver (Figure 2D). The underlying mechanism may carry out the lipogenesis and lipid-lowering biological process by decreasing the expression of *srebpf1* and *fasn* and increasing the expression of *pparα* and *pparγ* (Figures 4A,B). PPARα and PPARγ are expressed in numerous tissues, such as liver, adipose, and muscle tissue, playing a major role in regulating lipid metabolism. PPARα performs lipid degradation and FA utilization through mitochondrial or peroxisomal oxidation (Mate et al., 2017). PPARα ligands reduce very-low-density lipoprotein (VLDL) production and enhance the catabolism of TG-rich particles and hepatic elimination of excess cholesterol (Lefebvre et al., 2006). PPARγ is almost expressed in adipose tissue, and PPARγ ligands like PIG could reverse lipotoxicity by promoting subcutaneous fat mass expansion and lipid storage capacity (Cariou et al., 2012), enhancing TG *β*-oxidation and FAs utilization in the liver (Gross et al., 2017).

Nuclear factor erythrocyte 2-related factor 2(Nrf2) plays a pivotal role in defense against oxidative stress *via* induction of antioxidant response element (ARE)-driven genes in the cellular, such as hemeoxygenase-1(HO-1), and decreased the oxidant stress production ROS and MDA (Cuadrado et al., 2019). In NAFLD, oxidative and inflammation could accelerate the development of NASH, which is a more aggressive progression of the disease. Thus, induction of Nrf2 seemed to be promising in the prevention and treatment of NAFLD by anti-oxidation and reducing inflammation. HBA is a phenolic compound derived from herbs, and it shows a good preventive and therapeutic effect on chronic diseases related to oxidative stress, especially in central nervous system diseases, by inducing the expression of Nrf2 (Yu et al., 2013; Luo et al., 2017). Given that, to explore the role of HBA in oxidative stress on NAFLD, DCFH-DA staining revealed the ROS level in HCD induced larval zebrafish, we measured the ROS and MDA level with anti-oxidized SOD and HO-1. The results clarified that HBA could reduce fluorescence intensity and area (Figure 3A) as well as the ROS and MDA level (Figure 3B) and could also increase the activity of SOD and the level of HO-1, especially in the second process of the model (Figure 3C). This may be due to the anti-oxidation effect not being disturbed in the first process, as demonstrated by the SOD activity. The HO-1 level was considered with control in the HCD group, and the Nrf2 pathway was not interfered with. However, the anti-oxidation mechanism did not work in the second process, and HBA recovered the effect of anti-oxidation in NAFLD larval zebrafish by targeting the Nrf2-HO-1 pathway and reducing the expression of inflammatory cytokines (Figures 4C,D). Based on the effect of HBA on NAFLD larval zebrafish, we believe HBA may improve the lipid-induced oxidative stress by targeting the Nrf2 pathway.

Moreover, we further explored the underlying mechanism of HBA on NAFLD using FFA-inducing BRL-3A rat hepatocytes *in vitro*. The ability to uptake FFA and the level of oxidative stress was measured by Nile Red staining and DCFH-DA staining. The result showed that HBA could decrease the FFA uptake in BRL-3A hepatocytes and reduce the ROS level triggered by FFA. Nrf2 played a role to improve oxidation by nucleus transposition then promoting the expression of the downstream gene. Our result proved HBA facilitated Nrf2 nucleus transposition by immunofluorescent staining then increasing the expression HO-1 to weaken oxidative stress. Nrf2 Nucleus transposition is an essential process; this is followed by bonding with ARE to activate the downstream genes HO-1, GST, and CYPS and to promote the expression of antioxidase and Ⅱ phase detoxifying enzymes to perform the role of anti-oxidative stress.

## Conclusion

Taken together, the present study proved the role of HBA in reducing the lipid level and providing protection from lipid-mediating oxidative stress by modulating lipid metabolism and the Nrf_2_ pathway. HBA has shown efficiency in reducing the lipid levels during HCD inducement of the different NAFLD disease progression on the larval zebrafish model and FFA-induced BRL-3A cells. The potential mechanism of HBA is to mediate the lipogenesis of PPARα and the PPARγ pathway. Moreover, HBA could ameliorate oxidative stress by reducing ROS and MDA levels, increasing the activity of antioxidizes SOD and the level of HO-1. The intrinsic mechanism may act on the Nrf2 pathway by motivating the nuclear translocation of Nrf2, and it may activate the expression of the downstream gene HO-1. The results suggest that HBA has a potential efficacy in reducing lipid levels and improving oxidative stress on NAFLD, and it is therefore worthwhile to analyze this further as a potential therapeutic drug.

## Data Availability

The raw data supporting the conclusions of this article will be made available by the authors, without undue reservation, to any qualified researcher.
