# Supplementary figures and images for: P- Hydroxybenzyl Alcohol Alleviates Oxidative Stress in a Nonalcoholic Fatty Liver Disease Larval Zebrafish Model and a BRL-3A Hepatocyte Via the Nrf2 Pathway
Source: Front Pharmacol. 2021 Apr 12;12:646239. doi: 10.3389/fphar.2021.646239 (PMC8071996; doi:10.3389/fphar.2021.646239)

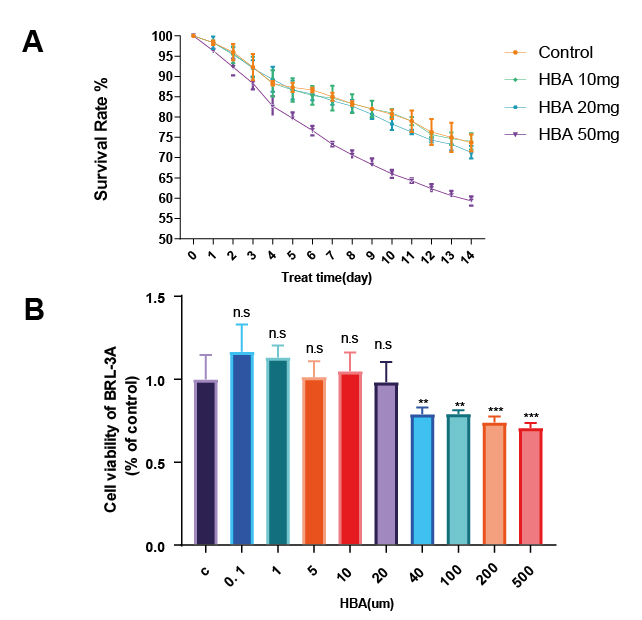

Supplement: Supplementary file 1 [file image1.jpeg]

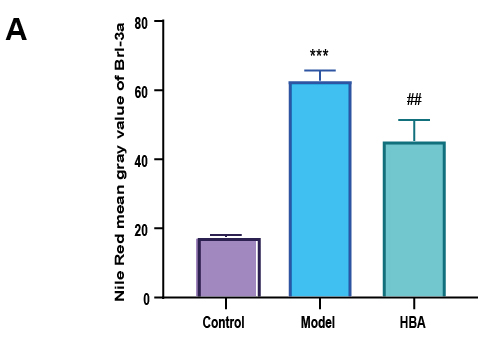

Supplement: Supplementary file 2 [file image2.jpeg]
