# Supplementary material for: P- Hydroxybenzyl Alcohol Alleviates Oxidative Stress in a Nonalcoholic Fatty Liver Disease Larval Zebrafish Model and a BRL-3A Hepatocyte Via the Nrf2 Pathway
Source: Front Pharmacol. 2021 Apr 12;12:646239. doi: 10.3389/fphar.2021.646239 (PMC8071996; doi:10.3389/fphar.2021.646239)
Supplement: Supplementary file 3 [file table1.docx]

**Table 1 Specific sequences of primers used in qRT-PCR**

| Gene name | Acceccion number | Forward primer (5’->3’) | Reverse primer (5’->3’) |
| --- | --- | --- | --- |
| *Danio rerio* |  |  |  |
| srebf1 | NM_001105129 | CATCCACATGGCTCTGAGTG | CTCATCCACAAAGAAGCGGT |
| fasn | XM_005169478 | ATCTGTTCCTGTTCGATGGC | AGCATATCTCGGCTGACGTT |
| pparab | NM_001102567 | CGTCGTCAGGTGTTTACGGT | AGGCACTTCTGGAATCGACA |
| pparg | NM_131467 | CTGCCGCATACACAAGAAGA | TCACGTCACTGGAGAACTCG |
| tnfa | NM_212859 | GCTTATGAGCCATGCAGTGA | TGCCCAGTCTGTCTCCTTCT |
| il1b | NM_212844 | TGGCGAACGTCATCCAAG | GGAGCACTGGGCGACGCATA |
| il6 | NM_001261449 | AGACCGCTGCCTGTCTAAAA | TTTGATGTCGTTCACCAGGA |
| keap1 | NM_182864.2 | CCAACGGCATAGAGGTAGTTAT | CCTGTATGTGGTAGGAGGGTT |
| nrf2 | NM_182889.1 | TTGTCTTTGGTGAACGGAGGT | CTCGGAGGAGATGGAAGGAAG |
| HO-1 | NM_001127516.1 | GCTCAACATCCAGCTCTTTGAGG | GACAAAGTTCATGGCCCTGGGA |
| Rattus norvegicus |  |  |  |
| srebf1 | NM_001276708.1 | ACTGCTGTAAAGATGTACCCGTCCG | GGCACTGGCTCCTCTTTGATTCC |
| fasn | NM_017332.2 | CTTTGTGAGCCTCACCGCCAT | ATGCCATCAGGTTTCAGCCCC |
| keap1 | NM_057152 | TGCTCAACCGCTTGCTGTATG | CCAAGTGCTTCAGCAGGTACA |
| nrf2 | NM_031789.2 | TTGTAGATGACCATGAGTCGC | TGTCCTGCTGTATGCTGCTT |
| HO-1 | NM_012580.2 | GTAAATGCAGTGTTGGCCCC | ATGTGCCAGGCATCTCCTTC |

| Gene name | Acceccion number (Danio rerio) | Forward primer (5’->3’) | Reverse primer (5’->3’) |
| --- | --- | --- | --- |
| *Danio rerio* |  |  |  |
| srebf1 | NM_001105129 | CATCCACATGGCTCTGAGTG | CTCATCCACAAAGAAGCGGT |
| fasn | XM_005169478 | ATCTGTTCCTGTTCGATGGC | AGCATATCTCGGCTGACGTT |
| pparab | NM_001102567 | CGTCGTCAGGTGTTTACGGT | AGGCACTTCTGGAATCGACA |
| pparg | NM_131467 | CTGCCGCATACACAAGAAGA | TCACGTCACTGGAGAACTCG |
| tnfa | NM_212859 | GCTTATGAGCCATGCAGTGA | TGCCCAGTCTGTCTCCTTCT |
| il1b | NM_212844 | TGGCGAACGTCATCCAAG | GGAGCACTGGGCGACGCATA |
| il6 | NM_001261449 | AGACCGCTGCCTGTCTAAAA | TTTGATGTCGTTCACCAGGA |
| keap1 | NM_182864.2 | CCAACGGCATAGAGGTAGTTAT | CCTGTATGTGGTAGGAGGGTT |
| nrf2 | NM_182889.1 | TTGTCTTTGGTGAACGGAGGT | CTCGGAGGAGATGGAAGGAAG |
| HO-1 | NM_001127516.1 | GCTCAACATCCAGCTCTTTGAGG | GACAAAGTTCATGGCCCTGGGA |
| Rattus norvegicus |  |  |  |
| srebf1 | NM_001276708.1 | ACTGCTGTAAAGATGTACCCGTCCG | GGCACTGGCTCCTCTTTGATTCC |
| fasn | NM_017332.2 | CTTTGTGAGCCTCACCGCCAT | ATGCCATCAGGTTTCAGCCCC |
| keap1 | NM_057152 | TGCTCAACCGCTTGCTGTATG | CCAAGTGCTTCAGCAGGTACA |
| nrf2 | NM_031789.2 | TTGTAGATGACCATGAGTCGC | TGTCCTGCTGTATGCTGCTT |
| HO-1 | NM_012580.2 | GTAAATGCAGTGTTGGCCCC | ATGTGCCAGGCATCTCCTTC |
